# Effectiveness of a novel diet in attenuation of clinical activity of disease in patients with ulcerative colitis: a randomized, clinical trial

**DOI:** 10.1038/s41598-024-64512-8

**Published:** 2024-06-14

**Authors:** Behnaz Narimani, Amir Sadeghi, Nasser Ebrahimi Daryani, Shabnam Shahrokh, Maryam Nilghaz, Maryam Ghods, Mahshad Shafiee, Mohammad Reza Shahparvari, Azita Hekmatdoost

**Affiliations:** 1 Department of Clinical Nutrition, National Nutrition and Food Technology Research Institute, School of Nutrition and Food Technology, Shahid Beheshti University of Medical Sciences, Tehran, Iran; 2 Gastroenterology and Liver Diseases Research Center, Research Institute for Gastroenterology and Liver Diseases, Shahid Beheshti University of Medical Sciences, Tehran, Iran; 3 Department of Gastroenterology, and Liver Diseases, Tehran University of Medical Sciences, Tehran, Iran; 4 Department of Clinical Nutrition and Dietetics, Faculty of Nutrition and Food Technology, Shahid Beheshti University of Medical Sciences, Tehran, Iran

**Keywords:** Ulcerative colitis, UC, IBD, Diet, EN, Nutrition, Mediterranean diet, Low-FODMAP diet, Gastrointestinal diseases, Nutrition disorders

## Abstract

Dietary intake plays a pivotal role in ulcerative colitis (UC) initiation and prognosis. The aim of this study was to investigate the effect of a combined Mediterranean, low-FODMAP diet accompanied with partial enteral nutrition (PEN) on clinical and para-clinical characteristics of patients with UC. Fifty patients with active mild to moderate UC were received either a combined diet or a regular diet for 6 weeks. Before and after the intervention, disease activity index, quality of life and some inflammatory and oxidative stress factors were measured using valid and reliable questionnaires and blood sampling. Disease activity index was significantly decreased in the combined diet group in comparison to control diet group (p = 0.043), and baseline data (p < 0.001). Moreover, the quality of life score increased significantly in the combined diet group compared to the control group, and the baseline data (p < 0.001). Serum level of high sensitive C-reactive protein (hs-CRP) decreased significantly in the combined group (p < 0.01), while it increased in the control group non-significantly. Serum total anti-oxidant capacity (TAC) changes were not statistically significant in two groups. This study indicates that this combination diet has the potential to be used as a safe and highly effective approach in patients with significant intestinal symptoms. Further clinical trial studies with different duration of intervention are needed to confirm these results.

*Trial registration*: The study was registered on IRCT.ir with registration number of IRCT20100524004010N38, on 25/04/2023.

## Introduction

Ulcerative colitis (UC) as a subtype of inflammatory bowel disease (IBD) is a life‐long idiopathic intestinal disorder with frequent relapsing and remitting inflammation of the colonic mucosa^1^. Patients usually have symptoms such as rectal bleeding, diarrhea, bloating, abdominal cramps and weight loss which in the active stage of the disease negatively affect the patient's quality of life and impose a large social and medical burden^2^. Epidemiologic studies have shown that the prevalence and incidence of UC is increasing in both developed and developing countries, with the highest rates of UC in Western countries^3^. Although the pathogenesis of this disease is not completely clear, a combination of environment, susceptible genes, inappropriate diet and immune responses have been considered to cause UC^4,5^. Existing medical therapies for UC, such as anti-inflammatory and immunosuppressive agents, while reducing the symptoms, have various side effects like abnormalities in eyes, bones, and gastrointestinal and liver function making them a big challenge in today’s gastroenterology^4,6^. For these reasons, it is important to find complementary therapies with low prices and high efficacy in the hope of improving the disease and reducing the symptoms. In recent years, dietary structure has received attention due to its major role in the clinical care of UC patients^7,8^. Moreover, patients with UC are interested in finding nutritional guidance to help improve their quality of life and relieve symptoms^9–12^.

Previous studies have suggested the beneficial roles for Mediterranean^13^, low FODMAP (fermentable oligosaccharides, disaccharides, monosaccharides, and polyols) diets^14^, and partial enteral nutrition (PEN)^15^ individually. The Mediterranean diet is one of the safe dietary patterns recommended by ESPEN (European Society of Clinical Nutrition and Metabolism) which recommends a high consumption of vegetables and fruits, unsaturated fatty acids (mainly from olive oil and nuts), dry pulses, dairy products and fish and low consumption of red meat and processed foods, rich in saturated fatty acids and simple sugars^16^. Previous studies showed that it can be useful for managing the symptoms of UC patients and improves the ratio between pathogenic microorganisms such as Firmicutes and Eschericha coli, and beneficial bacteria including *Bifidobacterium* and *Bacteroides fragilis*^17–19^. A prospective interventional study showed that a Mediterranean diet for about 6 months in patients with IBD improved their nutritional status and quality of life^13^. Moreover, randomized trial studies have shown that fermentable oligo-di-mono-saccharides and polyols (FODMAPs) can induce intestinal symptoms in both irritable bowel syndrome (IBS) and IBD^20,21^. Recent studies reported that intestinal symptoms improved in patients with IBD during a low-FODMAP diet compared to a control group^22–24^. Another strategy in management of IBD is the use of enteral nutrition (EN). Several studies have shown that enteral nutrition with a defined formula can alter the gut microbiota, improve nutritional status, and gut epithelium healing, which can be beneficial for clinical and mucosal recovery in adults and children with active Crohn’s disease and in serious cases of ulcerative colitis (UC)^15,25–28^.

Although the effects of these dietary changes on UC have been investigated separately in previous clinical studies, the results are inconsistent and their combined effect has not yet been investigated^29^. Therefore, we conducted a clinical trial to investigate the combination of a Mediterranean, low-FODMAP diet accompanied with supplemental formula consumption on the quality of life, and disease activity index in patients with mild to moderate active UC.

## Material and methods

### Trial design and participants

This study was an open-label, randomized, controlled, parallel-group, clinical trial. Patients with active mild to moderate UC whose UC diagnosis was previously confirmed by a gastroenterologist^30^ were recruited from two gastroenterology clinics in Tehran. The inclusion criteria included patients with more than 18 years old, free of cancer or other inflammatory, autoimmune, infectious and intestinal diseases. In addition, we excluded patients who were pregnant/lactating women, as well as those consuming some medications such as antihistamines, anticoagulants, calcium channel antagonists, or oral contraceptive drugs during the past month before starting intervention and those who were on special or vegetarian diets during the last three months before the intervention. Furthermore, participants who were on biological medications, changed the type and dosage of their medications in the last month were not included in the study, and those who had relapses that required hospitalization and changed the type and dosage of medications during the intervention or patients who did not want to continue the study protocol were identified and excluded from the study. The study protocol was approved by the ethics committee of the National Nutrition and Food Technology Research Institute (Ethics committee reference number IR.SBMU.NNFTRI.1402.001) and all participants signed a written informed consent form. The study was registered on IRCT.ir with registration number of IRCT20100524004010N38, on 25/04/2023.

### Intervention

We divided the participants into two groups, the combined diet group and the control group. All participants were given face-to-face dietary counseling by a dietitian for 45 to 60 min at the beginning and the end of the intervention. At the beginning of the study, participants in the combined diet group were given a six-week structured menu plan that included recipes and nutritional tips to follow the diet, as well as they received the formula powder required for 6 weeks along with its usage instructions. The menu plan was based on 75% the amount of calories calculating using the Mifflin formula needed to maintain weight based on the principles of Mediterranean, low-FODMAP diets and the remaining 25% of calories was provided by standard formulas (Fastmeal® standard). Diet adherence and events of treatment were followed up by the same nutritionist during weekly telephone counseling sessions. The patients who were assigned to control group received dietary recommendations for healthy eating (not menu plans).

### Clinical and dietary assessments

At the baseline and the end of the study, anthropometric data including weight (with light clothes to the nearest 0.1 kg), height (without shoes to the nearest 0.5 cm) were measured and BMI (weight (kg)/height (m^2^)) was calculated and a "general questionnaire" (including general information about age, gender, duration of the disease, smoking history, current medications, etc.) was completed by an expert interviewer. We used the "Inflammatory Bowel Disease Questionnaire-9 (IBDQ-9)"^31^ to assess the health-related quality of life and "Simple Clinical Colitis Activity Index Questionnaire (SCCAIQ)"^32^ to determine disease activity index. Furthermore, dietary intake data were collected for 3 days of 2 weekdays and 1 weekend day to assess the dietary intake of energy, macro/micronutrients of the patients, and their adherence to the administered diet. If patients did not follow the recommended diet for more than two days, they were excluded from the study.

### Measurements

Dietary intake data were analyzed by modified 4 nutritionists using national food composition tables^33^. IBDQ-9 was calculated for each participant's quality of life. The validity, reliability and high sensitivity of the IBDQ questionnaire have been proven in previous studies, and it is claimed that it has a significant correlation with clinical and colonoscopy results, for this reason it is known as the most utilized questionnaire for assessing the quality of life in patients with IBD in clinical and epidemiological studies^31^. This questionnaire contains 9 questions that examines various factors affecting patients' life such as the patient's satisfaction, energy level, fatigue, the frequency of postponing work, and intestinal symptoms such as stool frequency, gas excretion, flatulence and abdominal cramps during past two weeks. Each question has 7 items that are scored from 1 to 7, so the total score of questionnaire vary from 9 to 63. The higher the score, the better the quality of life^34^. Also, SCCAIQ score was rated to evaluate disease activity. The reliability of this questionnaire has already been confirmed and it is claimed that this questionnaire is closely related to the laboratory data^32^. This questionnaire has 6 questions and its total score is from 0 to 19, which the higher score indicates more severity the disease during past week.

### Sample size calculation

With a power of 80%, a type I error of 5% and detection of 4 score difference in mean SCCAIQ score, the sample size for each group was calculated as twenty one patients in this study^35^. Due to the potential loss of samples, 25 patients in each group were considered.

### Primary and secondary outcomes

The primary outcome was a significant reduction in SCCAIQ score. The primary analysis was intention to treat with all patients analyzed according to assigned treatment. Patients missing a SCCAIQ measurement were imputed as not achieving a sustained reduction. Secondary outcome measures were IBQ-9 score, high sensitive C-reactive protein in serum and total anti-oxidant capacity (TAC), and anthropometric variables.

### Biochemical measurements

After 12–14 h of fasting, blood samples were collected from the participants at the baseline, and the end of intervention. For separating plasma, the blood samples were first centrifuged, and the upper segments were collected in microtubes, and were frozen at − 80 °C for the next procedures. The plasma concentration of TAC was measured using a spectrophotometric method using Randox TAS (Randox Laboratories, Crumlin, UK) by an autoanalyzer (Model Alcyon 300; Abbott, Abbott Park, IL, USA). Plasma concentration of C-reactive protein (hs-CRP) was measured using the Kit produced by Diagnostic Biochem Canada Inc. using ELISA method.

### Statistical analysis

SPSS (version 26) was used for statistical analyses and P < 0.05 was considered statistically significant. We used the Shapiro-Wilks test to assess the normality of the data distribution. Chi-squared (χ2) test was used to compare the qualitative confounding variables and regarding quantitative variables, if their distribution was normal, paired t test was used to compare their mean in each group and t test was used to compare their mean between two groups, and if their distribution was not normal, the Wilcoxon test was used to compare them in each group, and the Mann–Whitney test was used to compare them between two groups. Also, in order to eliminate the effect of quantitative confounding factors, we performed covariance analysis.

For intention to treat analyses, we used a multiple imputation (MI) procedure based on Multivariate Imputation by Chained Equations (MICE). In the MI procedure, five imputed data sets were generated. We pooled the results of the five imputed data sets to obtain data estimates.

### Ethical approval

The study protocol was approved by the ethics committee of the National Nutrition and Food Technology Research Institute (Ethics committee reference number IR.SBMU.NNFTRI.1402.001) and all participants signed a written informed consent form. This study complies with the Declaration of Helsinki and was performed according to ethics committee approval.

## Results

**Figure 1:**
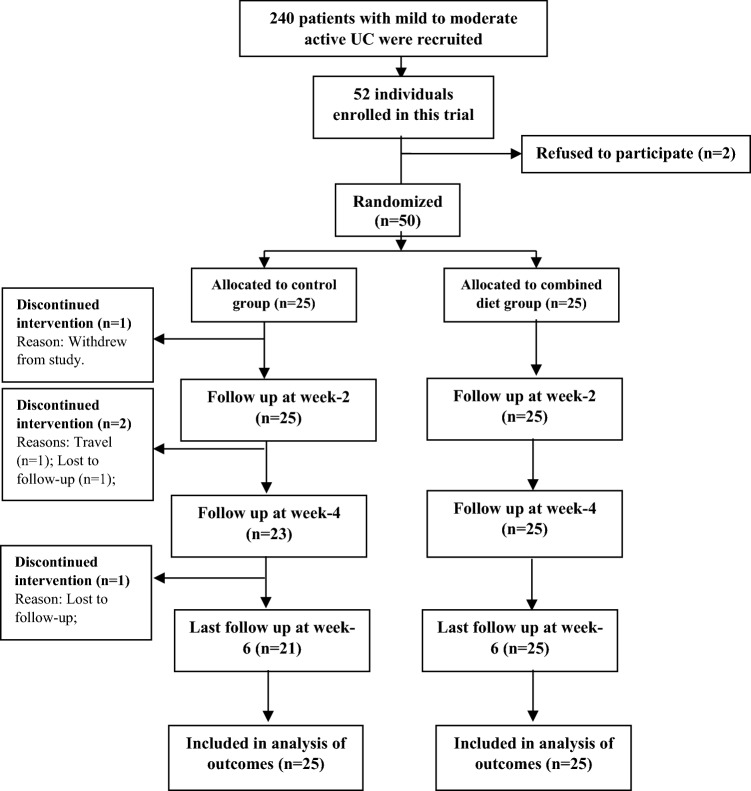
CONSORT flow diagram of the study participants.

**Figure 2:**
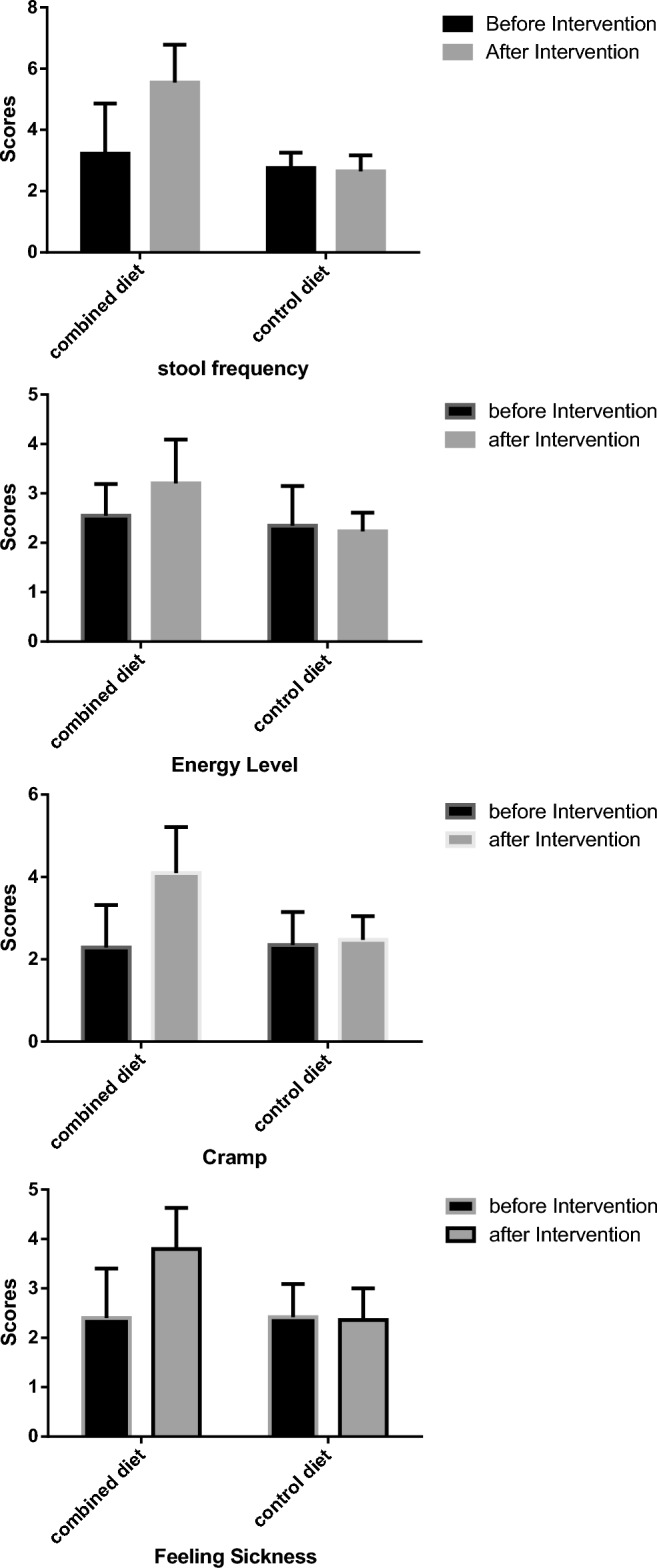

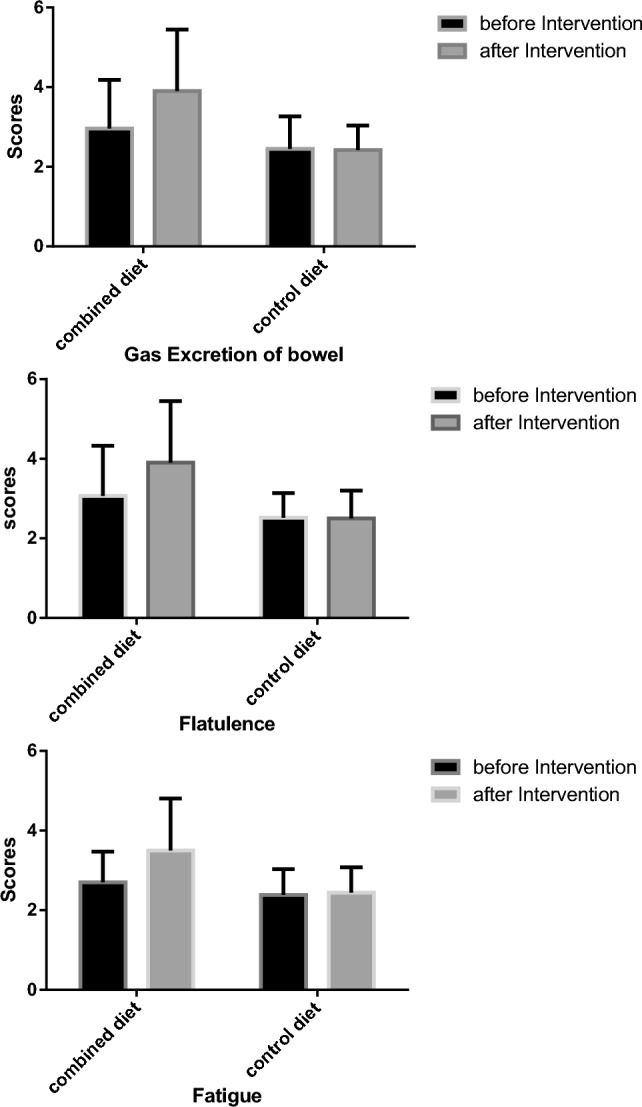
The main components of IBD related quality of life scores before and after the intervention.

Fifty patients with mild to moderate active UC were divided into two groups of the combined diet and the regular diet (Fig. 1). As it is shown in Table 1, there was no significant difference between the groups based on sex (p = 0.087), smoking (p = 0.856), disease duration (p = 0.307), age (p = 0.377), and medications (p > 0.05). Also, before and after the study, there was no significant difference between the groups and in each group in terms of weight and BMI (Table 2). Participants were similar in the case of their medications in two groups, and during the study. Dietary intakes data are shown in Table 3. The groups did not have significant differences in the case of dietary intake of energy, macronutrients and micronutrients before and after the intervention, except for poly-unsaturated fat and fiber, which were significantly lower in the combined diet group before the intervention; however, there was no significant change in each group, before and after the study. Quality of life, and disease activity index of patients before and after the intervention are shown in Table 4. There was no significant difference between the groups IBDQ score at the baseline; however, Mann–Whitney test showed that IBDQ score in the combined diet group was significantly higher than the regular diet group after 6 weeks of intervention (p =  < 0.001). Also, no significant difference was observed in patients quality of life factor such as fatigue score (p = 0.137), energy level score (p = 0.366) and feeling sickness score (p = 0.806), and intestinal symptoms such as stool frequency score (p = 0.689), gas excretion score (p = 0.233), flatulence score (p = 0.140) and cramps score (p = 0.685) at the baseline; however, these scores increased significantly in the combined group (p < 0.001 except for fatigue score (p = 0.004), gas excretion score (p = 0.001) and flatulence score (p = 0.003)) (Fig. 2). In addition, the two groups were statistically similar in disease activity score at the beginning of the intervention (p = 0.094); although it decreased significantly in the combined diet group at the end of study. As it is shown in Table 5, serum level of hs-CRP decreased significantly in the combined group (p < 0.01), while it increased in the control group non-significantly. Serum TAC increased in the combined diet group, and declined in the control group; however, these changes were not statistically significant.

**Table 1:** Individual characteristics of participants at the baseline.

	Combined group (n = 25)	Regular group (n = 21)	P*
Sex number (%)			0.08
Male, n (%)	10 (37%)	13 (61.9%)	
Female, n (%)	17 (63%)	8 (38.1%)	
Current smoker, n (%)	1 (3.7%)	1 (4.8%)	0.85
Age (year)	34.88 ± 9.53	39.76 ± 12.46	0.37
Duration of disease (year)	5.53 ± 7.76	5.71 ± 6.17	0.30
Patients on immuno-suppressants n (%)	9 (36%)	7 (33%)	0.58
Patients on 5-aminosalicylic acid n (%)	22 (88%)	19 (90%)	0.42
UC subtype n (%)			
Proctitis	3 (12%)	3 (14%)	0.82
Left sided colitis	12 (48%)	10 (47%)	
Pancolitis	10 (40%)	8 (38%)	
Fecal calprotectin µg/g	121 (75–257)	182 (82–396)	0.50
Fecal calprotectin < 150 µg/g n (%)	15 (60%)	11 (52%)	0.44
Serum albumin g/dl	3.5 ± 0.2	3.6 ± 0.2	0.98

Data are presented as mean ± standard deviation.

*chi-square test/Mann–Whitney test.

**Table 2:** Anthropometric indices of participants before and after the study.

	Before	After	P*
Weight (kg)			
Combined diet	70.61 ± 16.20	69.70 ± 16.68	0.228
Regular diet	67.73 ± 12.80	68.84 ± 12.89	0.793
P value (between group)	0.509	0.859	
BMI (kg/m^2^)			
Combined diet	24.98 ± 4.98	25.15 ± 5	0.220
Regular diet	24.42 ± 3.50	24.44 ± 3.62	0.641
P value (between group)	0.666	0.617	

Data are presented as mean ± standard deviation.

*Independent-sample T test/paired-sample T test.

**Table 3:** Dietary intake of participants before and after the study.

	Before	After	P*
Energy (Kcal)			
Combined diet	1669.97 ± 419.60	1647.23 ± 536.57	0.502
Regular diet	1475.10 ± 348.57	1583.18 ± 432.26	0.070
P** (between group)	0.104	0.481	
Protein			
Combined diet	52.62 ± 9.10	55.06 ± 17.06	0.279
Regular diet	54.82 ± 9.27	55.58 ± 7.15	0.295
P** (between group)	0.403	0.901	
Carbohydrate			
Combined diet	185.64 ± 59.65	188.89 ± 76.76	0.450
Regular diet	203.18 ± 51.70	194.16 ± 53.14	0.564
P **(between group)	0.306	0.799	
Total fat			
Combined diet	53.48 ± 11.24	52.55 ± 17.59	0.983
Regular diet	55.18 ± 11.98	51.08 ± 13.61	0.544
P** (between group)	0.625	0.767	
Fiber			
Combined diet	13.12 ± 3.34	15.84 ± 9.31	0.411
Regular diet	16.78 ± 3.53	15.11 ± 4.08	0.059
P** (between group)	0.001	0.735	
Saturated fat			
Combined diet	17.71 ± 6.00	14.61 ± 6.92	0.015
Regular diet	16.91 ± 4.89	16.02 ± 4.12	0.752
P** (between group)	0.636	0.431	
Mono-un saturated fat			
Combined diet	19.55 ± 6.55	20.84 ± 6.45	0.198
Regular diet	19.93 ± 8.33	19.15 ± 6.69	0.904
P** (between group)	0.863	0.419	
Poly-unsaturated fat			
Combined diet	14.34 ± 4.32	13.96 ± 4.34	0.581
Regular diet	17.20 ± 4.32	14.22 ± 5.28	0.077
P** (between group)	0.033	0.863	
Omega-3			
Combined diet	0.72 ± 0.53	0.59 ± 0.46	0.233
Regular diet	0.75 ± 0.31	0.57 ± 0.23	0.117
P** (between group)	0.415	0.872	
Omega-6			
Combined diet	8.26 ± 4.12	8.89 ± 3.86	0.192
Regular diet	9.68 ± 6.31	6.44 ± 5.22	0.169
P** (between group)	0.367	0.098	
Cholesterol			
Combined diet	184.56 ± 46.65	156.74 ± 118.4	0.256
Regular diet	155.08 ± 52.51	157.03 ± 51.71	0.494
P** (between group)	0.051	0.251	
Vitamin C			
Combined diet	58.59 ± 30.45	44.60 ± 27.98	0.065
Regular diet	45.29 ± 21.66	49.63 ± 16.68	0.547
P** (between group)	0.110	0.486	
Vitamin E			
Combined diet	15.25 ± 6.36	14.56 ± 5.03	0.101
Regular diet	14.77 ± 3.28	12.71 ± 5.57	0.194
P** (between group)	0.740	0.272	
Selenium			
Combined diet	73.15 ± 35.80	75.17 ± 45.26	0.460
Regular diet	54.80 ± 27.44	76.06 ± 42.54	0.153
P** (between group)	0.101	0.979	
Zinc			
Combined diet	10.31 ± 4.53	7.95 ± 3.85	0.010
Regular diet	8.14 ± 2.06	8.22 ± 1.96	0.841
P** (between group)	0.126	0.211	

Data are presented as mean ± standard deviation.

*Paired-sample T test or Wilcoxon test, **independent t test/Mann–Whitney test.

**Table 4:** Quality of life score and disease activity index of participants before and after the study.

	Before	After	P*
IBDQ score			
Combined diet	28.70 ± 5.40	38.55 ± 5.69	< 0.001
Regular diet	25.81 ± 2.83	26.05 ± 3.25	0.849
P** (between group)	0.068	< 0.001	
SCCAIQ score			
Combined diet	7.14 ± 2.97	5.50 ± 2.60	0.001
Regular diet	5.66 ± 2.98	4.04 ± 2.22	0.003
P** (between group)	0.094	0.043	

Data are presented as mean ± standard deviation.

*Paired-sample T test or Wilcoxon test, **independent t test/Mann–Whitney test.

**Table 5:** Inflammatory and total anti-oxidant capacity (TAC) factors in two groups, before and after Intervention.

Variable	Combined diet group (mean ± standard deviation (range))		Control group (mean ± standard deviation (range))		*P value
	Before intervention	After intervention	Before intervention	After intervention	
hs-CRP (ng/mL)	4767 ± 2258 (2502–7027)	2585 ± 1793 (790–4378)**	3159 ± 2418 (735–5575)	3537 ± 2345 (1185–5890)	≤ 0.001
TAC (U/mL)	1.87 ± 0.35 ( 1. 48–2.24)	2.1 ± 0.26 ( 1.68–2.35)	2.08 ± 0.41 (1.48–2.77)	1.72 ± 0.65 ( 0.99–2.65)	0.94
Fecal calprotectin	121 ± 82 (75–257)	108	182 ± 96 (82–396)		

The values are reported as mean value ± standard deviation and range.

*P value for efficiency of the combined diet using Mann–Whitney Test.

**The differences compared with the beginning of the research using Wilcoxon test, P value < 0.01.

No adverse effect was reported in any participant during the study.

## Discussion

This study is the first clinical trial to evaluate the effects of combined Mediterranean, low-FODMAP diets accompanied with supplemental enteral nutrition on quality of life, disease activity index, and some inflammatory and oxidative stress factors in patients with UC. Our results have shown that the implementation of our combined diet for 6 weeks improved the quality of life and related factors such as fatigue, energy level and feeling sick as well as intestinal symptoms such as stool frequency, gas excretion, flatulence and cramps. It also reduced the inflammatory factor of hs-CRP, and severity of the disease suggesting that this diet can be considered as a safe and beneficial approach in patients with active mild to moderate UC.

The importance of this study is obvious because previous studies have shown the role of diet in pathogenesis of UC. Previous studies have suggested an urgent need to design high-quality dietary studies in order to use diet therapy as a part of disease treatment to reduce patients ‘ need for medications, which leads to lower cost of treatment, and lower side effects of medications^36^. On the other hand, some studies have shown the beneficial effects of Mediterranean^13^, gluten-free^37^, lactose-free^38^, low-FODMAP diets^14^, and EN^15,25,39^, separately, on amelioration of UC activity. Although some features of low FODMAP diet and gluten free, lactose free diets are similar, Mediterranean diet has a different characteristic, which made this combination somehow complicated. Thus, we designed a personalized weekly dietary menu for every participant, and followed them regularly to answer their possible questions. Interestingly, participants followed the combined diet completely by the end of the study, which might be due to amelioration of their symptoms.

This combined diet could reduce both disease symptoms, and inflammation, as indicated by reduction of both disease symptoms, and blood hs-CRP. These beneficial effects might be due to the synergistic effects of each dietary protocol. Low FODMAP, gluten free, and lactose free diets reduce the symptoms of patients with UC due to low content of fermentable carbohydrates, resulting in low amount of gas production in the lumen^14,21,40^. EN can reduce both symptoms and intestinal inflammation due to its low bacteria content, high n-3 fatty acids, and low fermentable carbohydrates^15,25^. Mediterranean diet can reduce inflammation because of its high content of fresh fruits and vegetables, high quality oils, and low amount of sugars and saturated fats^13^.

Previous studies reported that low FODMAP diet induces promising effects on bloating and flatulence in UC patients^24^; however, there was a concern about the effects of this diet on beneficial gut bacteria, and gut inflammation. Thus, combination of low FODMAP diet with Mediterranean style, while adding enteral nutrition could overcome this concern, leading to even reduction in inflammation. This effect might be due to the beneficial components of Mediterranean diet, and enteral nutrition on gut microbiota, which results in reduction of gut permeability, and decreasing inflammation^13,25^.

Mediterranean diet is rich in fruits and vegetables, olive oil, and low in red and processed meat, which makes it somehow similar to anti-inflammatory diet. Our results are in line with previous studies^41,42^, which have found that anti-inflammatory diet including the increased intake of anti-inflammatory foods combined with a decreased intake of pro-inflammatory foods are associated with microbial changes in UC patients and prevent the disease risk, while modifying the gut microbiota. Thus, our idea for combination of low FODMAP diet with Mediterranean style and enteral nutrition was a successful thought to find the optimum diet for these patients.

This study has several strengths; pilot controlled trials are important to test early efficacy signals and the feasibility of a dietary intervention. This is the first clinical trial to assess the effects of a novel diet on disease activity and inflammation in patients with UC. No drop-out in the intervention group confirms the feasibility and effectiveness of this diet.

The limitations of our study lie in its evaluation methods, because invasive methods such as colonoscopy and tissue biopsy were not used to measure the severity of the disease due to ethical issues, although our questionnaires have been shown to be a proper tool for assessment of severity of the disease^32^. Moreover, we could not assess the variations in gut microbiota in this study due to limitation of the study fund. In this study, most patients claimed that the diet could reduce their intestinal symptoms. Since diets vary by geography, cultures, and other factors, the generalizability of these results to other populations is unknown.

In conclusion, we have shown that adherence to a combined Mediterranean, low-FODMAP diets accompanied with supplemental formula can improve disease severity index and quality of life, and inflammation in patients with active mild to moderate UC. These results are promising and can be a guide for further clinical trials with different durations to confirm our results.

### Supplementary Information

Supplementary Information.

## Data Availability

The data underlying this article will be shared on reasonable request to the corresponding author.
