# Supplementary material for: Effectiveness of a novel diet in attenuation of clinical activity of disease in patients with ulcerative colitis: a randomized, clinical trial
Source: Sci Rep. 2024 Jun 14;14:13791. doi: 10.1038/s41598-024-64512-8 (PMC11178895; doi:10.1038/s41598-024-64512-8)
Supplement: Supplementary file 1 — Supplementary Information. [file 41598_2024_64512_MOESM1_ESM.docx]

**A sample menu of the participants**

Breakfast: 60 gram gluten free bread, 60 gram hard cheese, one walnut, one tomato

Snack: One banana, a glass of lactose free milk

Lunch: 10 Tbsp. of cooked rice, 90 gram chicken or fish, 100 gram cooked green vegetables or carrot, 1 Tbsp. olive oil

Snack: a glass of orange juice, 30 grams of walnut

Dinner: 10 Tbsp. of cooked rice, 90 gram chicken or fish, 100 gram cooked green vegetables or carrot, 1 Tbsp. olive oil

Snack: a glass of lactose free milk or yogurt
